# Flat Detector CT with Cerebral Pooled Blood Volume Perfusion in the Angiography Suite: From Diagnostics to Treatment Monitoring

**DOI:** 10.3390/diagnostics12081962

**Published:** 2022-08-13

**Authors:** Thijs van der Zijden, Annelies Mondelaers, Maurits Voormolen, Tomas Menovsky, Maarten Niekel, Thomas Jardinet, Thomas Van Thielen, Olivier D’Archambeau, Paul M. Parizel

**Affiliations:** 1 Department of Radiology, Antwerp University Hospital (UZA), 2650 Edegem, Belgium; 2 Department of Medical Imaging, Algemeen Ziekenhuis KLINA, 2930 Brasschaat, Belgium; 3 Research Group mVision, Faculty of Medicine and Health Sciences, University of Antwerp (UA), 2610 Wilrijk, Belgium; 4 Research Group Translational Neurosciences, Faculty of Medicine and Health Sciences, University of Antwerp (UA), 2610 Wilrijk, Belgium; 5 Department of Neurosurgery, Antwerp University Hospital (UZA), 2650 Edegem, Belgium; 6 Western Australian National Imaging Facility, The University of Western Australia (UWA), Royal Perth Hospital (RPH), Perth, WA 6000, Australia

**Keywords:** imaging of vascular diseases of the brain, hemodynamics in cerebrovascular conditions, flat-panel CT, interventional imaging, perfusion imaging

## Abstract

C-arm flat-panel detector computed tomographic (CT) imaging in the angiography suite increasingly plays an important part during interventional neuroradiological procedures. In addition to conventional angiographic imaging of blood vessels, flat detector CT (FD CT) imaging allows simultaneous 3D visualization of parenchymal and vascular structures of the brain. Next to imaging of anatomical structures, it is also possible to perform FD CT perfusion imaging of the brain by means of cerebral blood volume (CBV) or pooled blood volume (PBV) mapping during steady state contrast administration. This enables more adequate decision making during interventional neuroradiological procedures, based on real-time insights into brain perfusion on the spot, obviating time consuming and often difficult transportation of the (anesthetized) patient to conventional cross-sectional imaging modalities. In this paper we review the literature about the nature of FD CT PBV mapping in patients and demonstrate its current use for diagnosis and treatment monitoring in interventional neuroradiology.

## 1. Introduction

The absolute numbers of people affected by cardiovascular diseases, and by extension, cerebrovascular diseases are increasing [1,2]. In the wake of this, therapeutic interventions on neurovascular patients are on the rise as well, since ischemic stroke has become a potentially (partially) curable disease, if and when diagnosed and treated early enough [3]. For diagnosis, treatment monitoring and follow-up in the management of patients with neurovascular diseases, different anatomic and physiologic determinants of neuronal tissue, such as parenchyma, blood vessels, perfusion and viability of tissue (infarct versus penumbra), should be investigated [4]. Conventional, cross-sectional neuro-imaging modalities, i.e., multidetector computer tomography (MD CT) or magnetic resonance imaging (MRI), can provide adequate information about all the aforementioned aspects of brain tissue. However, they typically represent a two-tiered management approach for the acute patient, requiring cross-sectional diagnostic imaging before the therapeutic endovascular intervention can started. Significant time could be saved if these imaging modalities would be available in the angiography suite.

Thanks to the advent of flat panel imaging techniques, it has become possible to perform flat detector CT (FD CT) imaging in the angiography suite, rendering cone beam CT images. Several studies have demonstrated the value of FD CT techniques in interventional neuroradiology [5,6,7,8,9]. FD CT imaging can match the image quality of conventional MD CT regarding the intracranial blood vessels, enabling adequate and immediate decision-making in the angiography suite for therapeutic interventions. On the other hand, visualization of brain parenchyma by FD CT imaging yields images of significantly lower quality, compared to those acquired with the conventional cross-sectional imaging techniques. However, in clinical practice the quality of the FD CT images suffices for appreciation of significant intracranial hemorrhages, presence of hydrocephalus and assessing Alberta Stroke Program Early Computed Tomography (ASPECT) score in acute stroke [10,11,12]. During recent years, in addition to vessel imaging and visualization of brain parenchyma, it has become technically feasible to perform perfusion imaging in the angiography suite [13,14]. Importantly, it must be understood that angiographic perfusion imaging, which is commercially available in current angiographic systems, differs from conventional cross-sectional perfusion imaging based techniques, given that C-arm acquisitions suffer from lower temporal resolution. Calculation of dynamic perfusion im-aging metrics, e.g., time to peak (TTP), cerebral blood flow (CBF), mean transit time (MTT), is not possible on most C-arm systems used in clinical practice. On the other hand, blood volume mapping, in the form of neuro Parenchymal (or Pooled) Blood Volume (PBV), is possible by calculating the area under the time density curve acquired by rotational im-aging during steady state contrast injection [15]. In Table 1 a short overview of several aspects of angiographic FD CT PBV perfusion imaging compared to MD CT perfusion imaging is presented.

**Figure 1:**
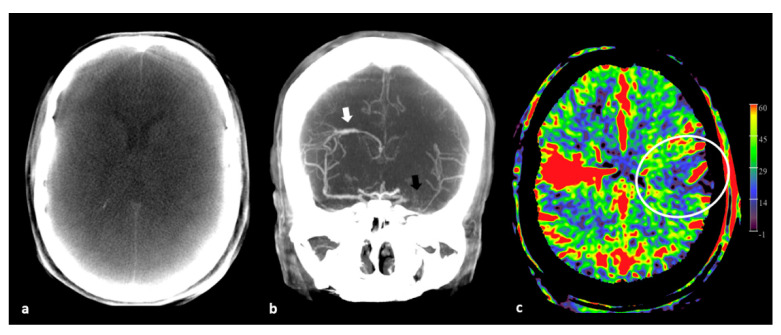
Three available maps by FD PBV imaging. (**a**) Axial maximum intensity projection (MIP) reformation of PBV mask run in an acute ischemic stroke (AIS) patient demonstrates no obvious signs of infarct. However, the image quality is rather low. (**b**) Coronal fill run MIP image in another AIS patient shows no filling of the left middle cerebral artery (MCA, black arrow) M1-segment. Collateral filling post-occlusion is nicely demonstrated. As an incidental finding, a developmental venous anomaly (DVA, white arrow) is noted. (**c**) A PBV axial MIP reformation of the same patient of (**b**) (at the level of the DVA) displays a territorial low PBV area (ellipsoid), corresponding with the perfusion deficit area caused by the MCA M1-segment occlusion.

The angiographic perfusion imaging can be performed by both intra-arterial contrast injection (selective or by pigtail catheter in the ascending aorta), and intravenous contrast injection. Furthermore, it is possible to use the non-contrast “mask” run and the contrast-enhanced “fill” run for rendering multiplanar reformations to visualize the brain parenchyma and vascular structures. In Figure 1 the mask, fill, and PBV mapping are il-lustrated.

Despite the limitation of not providing any dynamic perfusion parameters, FD CT PBV imaging does have potentially significant advantages when compared to dynamic perfusion imaging. By performing only two scan sweeps (mask and fill run), the risk of motion artefacts is reduced and total radiation exposure to the patient remains limited.

The nature of PBV values acquired during FD CT imaging in the angiography suite is not well-defined. Stille et al. (2019) described that FD CT provides similar Cerebral Blood Volume (CBV) values and reconstructed blood volume maps as CT perfusion in patients with cerebrovascular disease [16]. Struffert et al. (2015) showed that in a small group of arterial ischemic stroke (AIS) patients, PBV values correlated less with CBV values acquired with MRI [17]. Another research group reported that in a series of 26 patients with delayed cerebral ischemia (DCI) after aneurysmal subarachnoid hemorrhage, the acquired PBV values corresponded best with a “CBF weighted” CBV parameter compared to MRI perfusion [18]. In the same group of patients the time density curves (TDC) were analyzed and it was shown that the temporal characteristics varied significantly among individual patients [19]. They found that most TDC temporal characteristics approximated, but did not reach completely a contrast medium steady state in the cerebral vasculature for the duration of the scan.

In 2014, Fiorella et al. published the results of a prospective study of 56 patients with cerebrovascular ischemic disease, comparing conventional, dynamic CT perfusion imaging with FD CT PBV imaging [20]. They found that FD CT PBV maps were 100% sensitive and 81.3% specific to detect any CBV deficit, and 100% sensitive and 62.9% specific to detect any CBV deficit of greater than one-third of a territory. In patients with large vessel occlusion (LVO), they found a tendency to overestimate the presence and volume of PBV maps, causing a drop in specificity to 77.8% for any CBV deficit detection and to 55.6% for detection of any CBV deficit greater than one-third of a territory. For patients without LVO, the specificities were 85.7% and 77.8%, respectively. Interestingly, in 14 patients out of 56 patients, the FD PBV examinations were inadequate, mostly due to motion artefacts, improper patient positioning, and the presence of radiopaque objects in the scan field. The failed examinations were attributed to inexperience and lack of routine at the time of scanning, and therefore considered to be preventable. It only serves to emphasize that implementation of this technique in daily routine requires a certain level of experience of the angiographic team.

A systematic review by Stille et al. (2019) assessed the diagnostic accuracy of FD CT PBV perfusion imaging [16]. Performing a proper meta-analysis was not possible, due to the quality of the studies with large heterogeneity of study populations and designs. Most publications represented feasibility studies, focusing on scanning and contrast protocols. Based on 11 studies, comparing angiographic PBV values with CBV values acquired with conventional CT perfusion (CTP), and 5 studies, comparing PBV values with MRI perfusion (MRP) CBV values, they concluded that FD CT renders similar CBV values and volume maps as CTP in patients with cerebrovascular disease. However, the authors stated that uncertainty remains about the diagnostic accuracy of FD CT perfusion imaging, due to its inferior specificity compared to CTP and MRP, and so additional studies are required.

Regarding radiation dose exposure, Struffert et al. (2014) examined the effective dose delivered to patients, comparing angiographic flat detector applications with conventional MD CT applications, including FD CT PBV and MD CT perfusion imaging by using an anthropomorphic phantom [21]. They found that the effective dose was in comparable range for the various applications. Another dose measurement study by means of an anthropomorphic phantom by Chu et al. (2014) confirmed the finding of comparable effective dose between FD CT PBV imaging and MD CT perfusion imaging. In case of collimation during FD CT imaging (slab PBV, ranging from the sella turcica to high convexity), they found a drop of the effective dose of FD CT PBV imaging to about one-third that of MD CT perfusion imaging. The review by Stille et al. (2019) showed that in most reported imaging protocols of the examined studies a total effective dose of >2 mSv per acquisition was reached, based on the doses per frame as indicated by the respective angiographic system manufacturers [16]. Fiorella et al. (2014) measured radiation exposure for both techniques and observed similar values for FD CT PBV and MD CTP imaging [20].

In this article, a variety of current and potential applications of FD CT PBV imaging in interventional neuroradiology will be outlined.

## 2. Applications of FD CT PBV

### 2.1. Acute Ischemic Stroke

**Figure 2:**
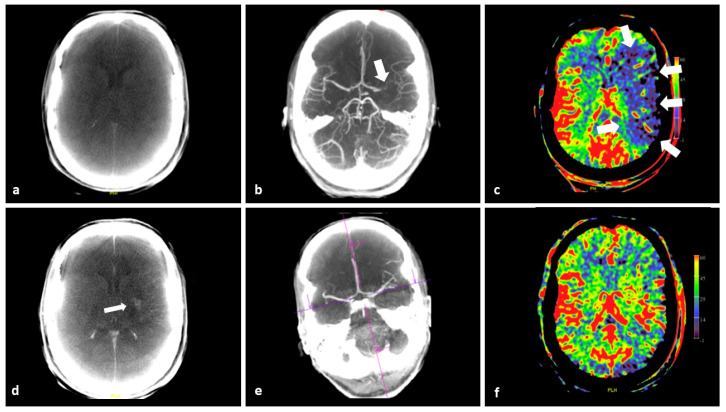
FD CT imaging in a 44-year-old female with AIS due to LVO, referred for EVT. (**a**) Pre intervention axial MIP reformation of the mask run demonstrates no hemorrhage. (**b**) Pre intervention axial MIP reformation shows occlusion at the level of M1-segment of the left MCA (arrow). (**c**) PBV MIP reformation features a perfusion deficit in the left MCA supply area (arrows). (**d**) Post EVT axial mask run MIP reformation demonstrates small area of contrast pooling in the left lentiform nucleus and the revascularized MCA territory (arrow). (**e**) Post EVT axial fill run MIP reformation confirms recanalization. (**f**) Post EVT axial PBV MIP reformation shows restoration of CBF with (almost) symmetrical PBV map.

Timely and adequate imaging is pivotal to ensure a good clinical outcome in the treatment of AIS patients [3,22,23]. The first step is to rule out hemorrhage and significant early ischemic brain parenchymal changes, in order to start appropriate treatment as quickly as possible. It is recommended to perform vessel imaging to select patients with LVO for endovascular thrombectomy (EVT) [24]. In current clinical practice, these imaging steps are performed by conventional MD CT or MRI as soon as possible. Nowadays, it is feasible to perform all the necessary imaging in the angiography suite thanks to the use of FD CT imaging, thus enabling a “one-stop-shop” approach in management of AIS patients [12,25]. Advanced imaging in the work-up of AIS patients within <6-h time interval from symptom onset to imaging should not delay recanalization treatment [24]. Therefore, it is not strictly recommended for selecting patients for EVT in <6 h. However, in late-presenting or wake up stroke patients, additional perfusion and/or MRI diffusion weighted imaging (DWI) is endorsed to appreciate the ratio of potentially salvageable brain tissue to already infarcted brain tissue [24]. If there is a significant time delay between initial stroke work up imaging and EVT, for instance because the patient requires transportation from the primary stroke center to a comprehensive stroke center, or in cases of significant alteration of the clinical status of the patient, additional imaging, including perfusion imaging, can be very useful [25]. FD CT PBV imaging not only provides perfusion PBV images, but also mask run images and fill run images, which can be used to detect brain parenchymal and vascular abnormalities, respectively, as demonstrated in Figure 2.

The use of PBV imaging in the setting of AIS has been examined in a few publications. Initial experiences with FD CT neuro PBV imaging in small groups of AIS patients showed good correlation of FD CT PBV deficits with MD CT perfusion CBV deficits [9,13]. It is reported that application of MD CT CBV mapping can be used for identification of LVO patients who may benefit from recanalization therapy [26,27,28,29]. Therefore, similar to CBV mapping in MD CT perfusion, with limitations, PBV mapping could be used in clinical routine for distinction between infarcted and potentially salvageable brain tissue.

A major point of criticism regarding the use of PBV mapping as a surrogate for conventional CBV mapping, is its questionable agreement with conventional CT or MRI perfusion CBV mapping. A study, comparing pre-procedural FD CT maps in 101 AIS patients referred for endovascular therapy (EVT), demonstrated that in 75.2% of cases, the PBV lesions matched with final infarct volumes at follow-up CT scans [30]. In the remaining 24.8% of the cases, they found a significant larger PBV volume at pretreatment scans than final infarct volume at follow-up imaging. The authors of this study concluded that PBV mapping might be useful for re-evaluation of potential infarct growth and collateral status prior to EVT after initial imaging but should not be used as the only criterion for patient exclusion. The conclusion of another study with 29 AIS patients, confirming overestimation of final infarct on angiographic CBV/PBV imaging in 25% of patients, was more pronounced, stating that FD CT PBV mapping should not be used for decision making for EVT at all [31].

A recent paper by Potreck et al. (2021) compared ASPECT scores obtained from pre-intervention FD CT PBV maps, with non-contrast MD CT images and MD CT perfusion CBV maps, and MD CT perfusion CBV maps with final infarct volumes at follow-up scans [32]. They found that unenhanced MD CT ASPECT scores outperformed both PBV ASPECT scores and CBV ASPECT scores in accuracy and reliability. Interestingly, they confirmed the presence of relevant infarct overestimation in PBV ASPECT scores compared to MD CT or MRI CBV ASPECT scores.

Another point of interest regarding the use of CBV mapping as a perfusion parameter for estimating infarct core size, is the fact that an increased or normal CBV does not always indicate salvageable brain tissue [33].

In summary, at this time, FD CT PBV imaging (mask run, fill run and PBV mapping) usage for a one-stop-shop approach in AIS patients referred for EVT is limited. In about 25% of cases, an overestimation of final infarct prediction based on FD CT PBV lesion volume is seen. PBV mapping is considered only useful as an additional perfusion deficit detection tool in LVO AIS patients. The exact nature of PBV mapping has not been established yet and its potential for infarct core size estimation is subject of debate. Therefore, further research seems justified to clarify its potential role in AIS treatment.

### 2.2. Tumor Embolization

The use of C-Arm based FD CT PBV imaging regarding pre- and post-tumor embolization has already been studied in the liver [34]. This imaging technique not only allows the detection of lesions in the liver amenable for chemo-embolization, but is also used for treatment monitoring. The presence of residual tumor perfusion on PBV maps could be a potential marker for mid-term tumor response in hepatocellular carcinoma (HCC).

In contrast to embolization of HCC, intracranial tumor embolization is usually performed in the pre-operative setting with the aim of reducing hemorrhagic risk during tumor resection [35]. Very few publications exist about FD CT PBV imaging in the setting of intracranial tumor embolization.

A semi-quantitative analysis of pre- and post-embolization neuro PBV scans in a series of 18 patients with hypervascular intracranial tumors was performed by Wen et al. [36]. After non-selective FD CT PBV scanning before and after tumor embolization, a semi-quantitative analysis was performed to examine the detection of perfusion deficits and the success of the embolization procedures. The authors observed a significant decrease in relative PBV values after embolization, compared to the values before embolization. Also, significant correlation between calculated post-embolic tumor perfusion indices with blood losses during surgery and with surgery times was demonstrated. The authors concluded that FD CT PBV imaging is a valuable method for evaluating hypervascular brain tumor perfusion and embolization efficacy.

**Figure 3:**
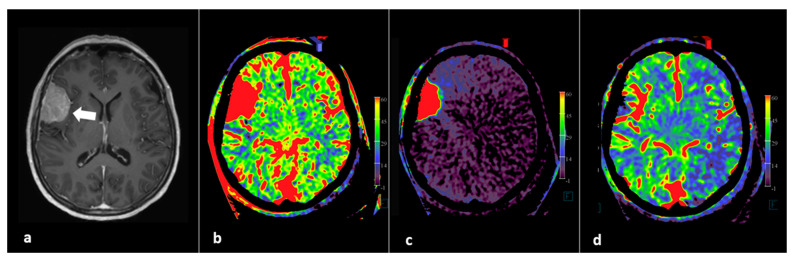
FD CT PBV imaging in a patient referred for pre-operative tumor embolization. (**a**) Axial T1-WI image shows right-sided perisylvic meningioma (arrow). (**b**) Pre embolization axial PBV MIP reformation after aortic root contrast demonstrating whole brain PBV imaging. (**c**) Pre embolization axial PBV MIP reformation with right external carotid artery (ECA) contrast injection demonstrates dominant dural vascular tumor supply. (**d**) Post embolization whole brain PBV axial MIP reformation illustrates successful tumor embolization.

In addition to whole brain neuro PBV imaging, selective contrast injection PBV imaging into tumor feeder vessels can be used to determine more precisely the potential benefit of the intended embolization [37]. Based on the FD CT imaging, the interventional neuroradiologist can immediately decide in the angiography suite, in agreement with the neurosurgeon, whether or not to perform a subsequent embolization procedure and, if applicable, select feeding arteries for embolization. FD CT PBV imaging pre- and post-tumor embolization scanning is demonstrated in Figure 3.

Summarizing, FD CT imaging allows exact volume PBV mapping by selective tumor and whole brain contrast injection. However, the exact value of qualitative or regional FD CT PBV measurements pre- and post-tumor embolization has not yet been decided and should be the subject of further research.

### 2.3. Cerebral Revascularization Procedures in Chronic Steno-Occlusive Disease

The mainstay for treatment of chronic steno-occlusive neurovascular diseases is to apply the best medical therapy in combination with proper life style changes [38,39,40].

Percutaneous transluminal angioplasty (PTA), by means of balloon dilatation and/or stenting, is a well-established and a widely applied therapeutic tool for treating stenotic neurovascular disease and should be considered in case of inadequate result of conservative treatment [41].

Surgical revascularization can be considered in cases, which are not amenable for conservative nor endovascular treatment, especially in moyamoya disease or syndrome. Depending on the clinical condition, a direct or indirect revascularization procedure can be performed. Direct revascularization (bypass) can be created from extracranial (EC-IC) or intracranial (IC-IC) donor arteries to intracranial acceptor arteries. Indirect revascularization can be done by constructing burr holes, combined with or without encephaloduroarteriosynangiosis (EDAS) [42,43,44,45]. Cranial bypass surgery can also be used in the setting of complex cerebral aneurysm treatment if parent vessel occlusion (PVO) is deemed necessary [46,47].

In addition to clinical evaluation, radiological investigation is needed for both monitoring of treatment efficacy and for detection of potential complications [48,49].

In case of stenting, FD CT imaging during or after stenting can be used to evaluate the patency and position of stents. Furthermore, FD CT imaging can detect hemorrhagic or thrombo-embolic complications at the spot in the angiography suite [6,7,8]. In clinical endovascular practice, appreciation of adequate flow restoration after stenting is sufficiently done by angiography. However, in a small proportion of patients with acute or chronic steno-occlusive disease of craniocervical arteries, cerebral hyperperfusion syndrome (CHS) or reperfusion damage can occur after successful revascularization therapy [50,51,52]. The reported incidence of CHS is up to 4.6% of carotid artery stenting (CAS) cases and in up to 50% in patients after bypass surgery and can result in seizures, focal neurological deficits, or intracranial hemorrhage. It is believed that this entity might be explained by impairment of local cerebral autoregulation, inflammation or, especially in patients after carotid endarterectomy, by autonomic dysfunction due to baroreceptor reflex breakdown or induction of free radicals by the surgical procedure, leading to increased cerebral blood flow [44,52]. Identification of patients at risk for developing CHS might allow early or preventive intervention to reduce neurological complications. In CAS patients, identified high risk factors were hypertension at baseline, treated carotid stenosis of >90%, and poor collateral blood flow [53]. Direct estimation of CBF can be done by conventional cross-sectional perfusion imaging techniques, i.e., single photon emission computed tomography (SPECT), CT perfusion and MRI perfusion, using either intravenously injected radio-isotopes or contrast agents [54]. Alternatively, the CBF can be measured without contrast media by using the MRI perfusion imaging Arterial Spin Labelling (ASL) technique [55]. In the angiography suite, it is possible to appreciate cerebral perfusion by means of quantitative or color-coded digital subtraction tomography or angiography (DSA), and C arm derived CBV or PBV imaging [56,57,58,59]. Prophylactic rigorous tension control by means of antihypertensive medication should be pursued in these patients. The duration of this antihypertensive therapy is not well established, due to the fact that CHS onset can differ between hours to days, risking unnecessary prolonged treatment [60].

In CAS, another therapeutic approach to high risk patients is “staged angioplasty”, which allows the patient’s brain to adapt gradually to the increased cerebral perfusion [52]. Perfusion imaging in the angiography suite before, during and/or after interventions might help to predict the risk for developing CHS, allowing a more tailored approach to these group of patients.

The role of FD CT PBV mapping in CAS has been explored by Fujimoto et al. (2018) [57]. In 30 patients pre- and post-stenting, regional C-arm CBV measurements were performed at cross-sections on standardized, symmetric regions of interest (ROIs) in the bilateral MCA territories. They found that postoperative C-arm CBV on the affected side was higher in patients who developed intracranial hemorrhage, with an increase in the postoperative CBV ratios. Postoperative CBV ratios were 1.03 ± 0.40 and 1.45 ± 0.68, and CBV ratio increase rates were 2.7 ± 24.0% and 28.5 ± 26.7% in the non-hemorrhagic and hemorrhagic group respectively. These differences were statistically significant (*p* < 0.01).

A recent study by Zhang et al. (2020) reported the application of peroperative Indocyanine Green-FLOW 800 Video angiography in a series of 62 moyamoya disease patients with 65 hemispheres treated by superficial temporal artery-MCA bypass surgery [61]. The peroperatively measured differences between pre- and post-operative peak CBV and regional CBF were significantly higher in the symptomatic group compared to the non-symptomatic group. Use of PBV imaging for prediction of CHS in this group of patients has not yet been investigated, but can hold promise.

**Figure 4:**
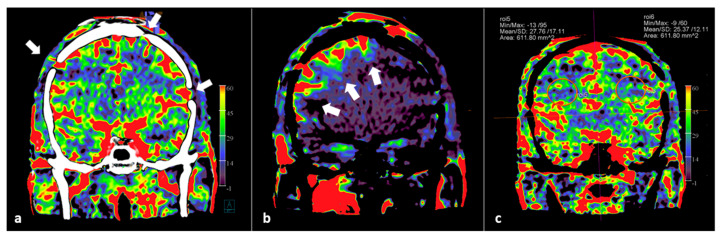
Selective and whole brain FD CT PBV imaging in a 29-year-old Asian male with moyamoya disease, treated by multiple burr holes surgery. Scanning was performed during aortic root injection (whole brain PBV imaging) and during selective ECA injection. (**a**) Coronal mask and PBV fusion MIP reformation demonstrates symmetrical PBV maps, and shows that multiple burr holes are present (white arrows). (**b**) Coronal PBV MIP reformation with selective right ECA contrast injection displays the blood supply to the right cerebral hemisphere (white arrows). (**c**) PBV value measurements in symmetrically plotted ellipsoid regions of interest (ROI) show symmetric PBV values.

Recently, we documented the value of PBV imaging in a small group of patients after bypass and burr hole surgery [62]. Besides whole brain PBV imaging with the possibility of PBV mapping, we demonstrated the potential of PBV mapping by selective contrast injection in the bypass arteries. We were able to precisely delineate the effect of individual bypass arteries, or the efficacy of dural artery recruitment after burr hole surgery. In Figure 4, the use of FD CT PBV scanning with selective contrast injection besides whole brain FD CT PBV imaging is demonstrated in a moyamoya disease patient treated with bilateral multiple burr holes surgery.

In summary, FD CT PBV imaging by means of selective single artery contrast injection and whole brain contrast injection allows the evaluation of the impact of revascularization procedures in patients with chronic steno-occlusive disease both during the procedure and in long-term follow-up.

### 2.4. Test Occlusion

In selected cases, parent vessel occlusion (PVO) can be applied as a safe and effective treatment option in complex, large artery aneurysms, post-traumatic arterial lesions or skull base tumors [63,64,65]. In such patients, especially in case of intended carotid artery occlusion, temporary balloon test occlusion (BTO) is used to evaluate the capability of a patient to endure the vessel occlusion. During test occlusion repeated clinical tests are performed, searching for signs of insufficient collateral blood flow to the tested hemisphere. In addition to clinical testing, conventional DSA is used for assessment of synchronicity in cortical venous filling (<2 s delay in the tested hemisphere) [66]. Nevertheless, a substantial part of the patients will suffer from delayed ischemic complications, despite passing the BTO clinically and angiographically [67]. Additional FD CT PBV imaging during test occlusion can yield more information about the suitability of PVO by calculating regional PBV values in different topographical areas of the brain, and comparing them with those derived from the non-affected contralateral hemisphere, especially combined with parametric color coding or plotted 2D time-density curves of DSA images. In a well-tolerated test occlusion, the goal should be to find normal PBV values without asymmetry. In theory, in case of elevated CBV or PBV values in the affected cerebral hemisphere, the brain is already trying to maximize its blood supply through reactive vasodilatation. Although no clinical symptoms might be present, the patient is at risk for delayed ischemic complications after PVO. In case of lowered CBV or PBV, the patient will show clinical symptoms already during test occlusion.

Yang et al. (2015) reported a case series of 10 BTO patients with hypotensive challenge [68]. Venous filling symmetry was evaluated by means of color coding of conventional DSA images. Additionally, angiographic FD CT CBV mapping was performed before and during BTO. Quantitative CBV values of regions of interests (ROIs) from affected and unaffected hemispheres were determined with calculation of relative CBV (rCBV) values. They observed in 7 of 10 patients good perfusion symmetry during BTO. Two patients failed for BTO, demonstrating significantly low FD CT CBV values at the affected hemispheres as well. Interestingly, one patient passed the BTO procedure clinically and angiographically, but showed hyperperfusion areas at FD CT CBV mapping, potentially indicating vascular insufficiency after possible PVO.

**Figure 5:**
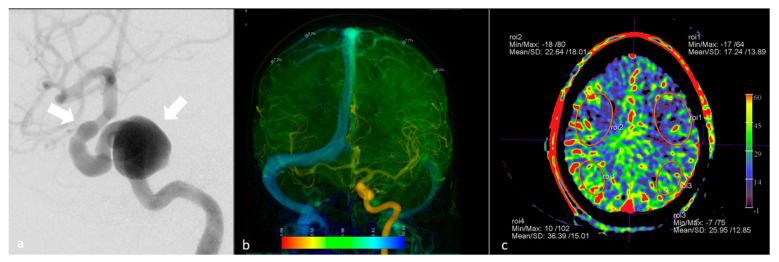
FD CT PBV imaging during test occlusion in a 57-year-old female with internal carotid artery aneurysms. (**a**) Depiction of the aneurysms (white arrows) on a lateral view DSA image. (**b**) Color-coded DSA image in anteroposterior view during test occlusion shows almost synchronous filling of the cortical veins, indicating the patient’s potential tolerance to PVO. (**c**) PBV MIP reformations can be utilized for plotting multiple ROIs in both hemispheres in a standardized fashion.

Figure 5 demonstrates a patient undergoing a neurological and angiographic test occlusion, including FD CT PBV imaging.

Another study about the use of FD CT PBV imaging during BTO was performed by Ikemura et al. (2020) [69]. A total of 12 patients underwent both SPECT and FD CT PBV imaging during BTO. The calculated mean interhemispheric ROI ratios for both SPECT CBF and FD CT PBV were unremarkable. In two patients, who developed subtle, late presenting neurological symptoms, a statistically significant decrease of both SPECT CBF and FD CT PBV in the affected hemisphere was noted. No increase of SPECT CBF or FD CT PBV was noted in tested hemispheres.

In conclusion, FD CT PBV imaging can help in decision taking in equivocal BTO cases. The potential role of FD CT PBV imaging for selecting patients for PVO in the setting of BTO should be further clarified, as current literature is scarce.

### 2.5. Aneurysm, Arteriovenous Malformation and Arteriovenous Fistula Embolization

C-arm cone beam CT imaging in the setting of cerebral aneurysm treatment is already widely used. FD CT imaging offers excellent image quality to ascertain the correct positioning of intravascular and/or intrasaccular devices during or after embolization procedures. In addition, flat panel CT imaging is capable of detecting hydrocephalus, hemorrhage and cerebral edema while the patient is on the angiography table, potentially delivering a significant positive impact on the clinical outcome [6,7,8,9].

In radiological follow-up after embolization of cerebral aneurysms, the use of angiographic C-arm CT with intravenous contrast injection can be implemented, thereby reducing the need for conventional DSA [70,71].

FD CT perfusion imaging in the angiography suite can facilitate detection of potential thrombo-embolic or vasospasm complications during embolization procedures.

**Figure 6:**
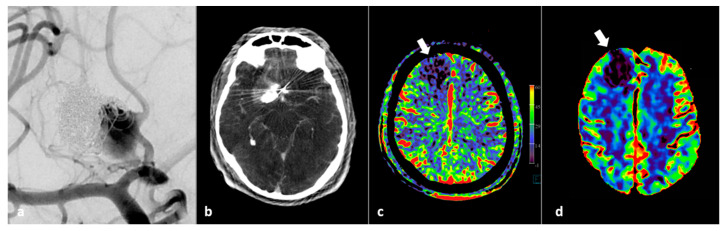
FD CT PBV imaging in a 46-year-old patient with thrombo-embolic and hemorrhagic complications during flow diverter placement. (**a**) AP view DSA image pre-intervention shows a significant neck remnant after previously coiled pericallosal aneurysm. (**b**) FD CT axial MIP reformation after occurrence of complications shows local hematoma and contrast. (**c**) Post PVO FD CT imaging shows right frontal residual wedge shaped perfusion deficit (white arrow). (**d**) Follow-up conventional MD CT CBV perfusion confirms local perfusion deficit.

Figure 6 illustrates the use of FD CT imaging for monitoring of occurrence of an endovascular complication.

**Figure 7:**
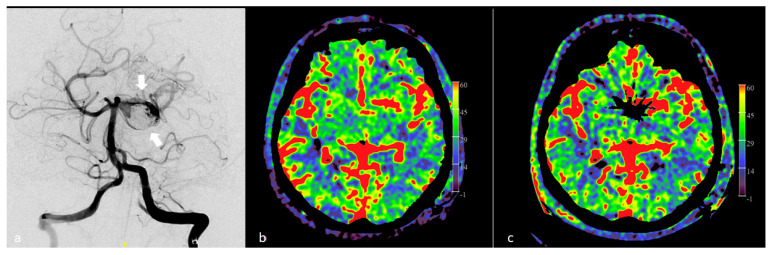
Intraprocedural FD CT PBV imaging in a case of PVO due to insufficiently treated dissecting P1–P2 aneurysm by stent assisted coiling. (**a**) DSA in AP view demonstrates the partially coiled dissecting aneurysm (white arrows). (**b**) Pre PVO FD CT PBV mapping demonstrates symmetric PBV maps. (**c**) Post PVO FD axial CT PBV MIP reformation shows no asymmetry in PBV values, suggesting the patient’s toleration to the PVO.

In addition, in case of considering PVO, the perfusion of the affected brain areas can be appreciated, potentially indicating additional bypass surgery. Figure 7 demonstrates the use of FD CT PBV imaging in a patient with left-sided dissecting P1–P2 posterior cerebral artery aneurysm, previously insufficiently treated by stent assisted coiling, treated by PVO.

Publications about perfusion imaging in patients with arteriovenous malformations (AVM) or arteriovenous fistulas (AVF) are scarce and to date no perfusion method can be considered as a gold standard. However, the use of steady state FD CT PBV mapping in patients with brain AVMs has been investigated [72]. The FD CT PBV data obtained in 5 patients with brain AVMs were compared to perfusion data obtained with conventional and ASL MRI perfusion imaging. It was found that FD CT PBV perfusion data were very variable and correlated best with CBF. The authors concluded that FD CT PBV mapping is inadequate for evaluation of brain AVMs. It appears that the required steady state contrast status during scanning is difficult to obtain, rendering highly “flow affected” PBV images.

**Figure 8:**
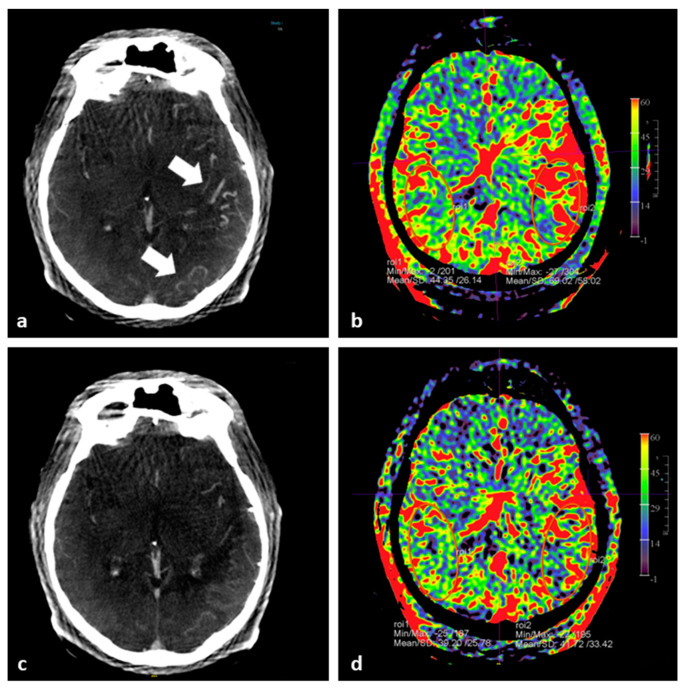
FD CT PBV imaging in DAVF treatment. (**a**) Axial FD CT fill run MIP reformation before treatment shows clearly the dilated and congested cortical cerebral veins at the right (white arrows). (**b**) Corresponding axial pretreatment PBV MIP reformation demonstrates elevated PBV values compared to the right side. (**c**) Axial fill run MIP reformation after partial embolization exhibits a clear diminishing of the cerebral venous congestion. (**d**) Post embolization axial PBV image shows symmetrical PBV values. The situation was accepted for the time being and the patient underwent uneventful a second embolization session 6 weeks later with complete occlusion of the DAVF.

On the other hand, FD CT PBV imaging after embolization might be beneficial to monitor the treatment effect and potential complications. For instance, in case of partial embolization of a high grade AVF, FD CT PBV imaging may direct the endpoint of an embolization procedure by appreciating the impact of (partial) embolization on cerebral venous congestion. In Figure 8, the use of FD CT PBV imaging during treatment of a type 3 dural AVF (DAVF) is demonstrated.

In summary, FD CT PBV imaging enables on the spot evaluation of potential hemorrhagic and thrombo-embolic complications, and assessment of (partial) embolization effect on brain hemodynamics during interventional neuroradiological procedures.

### 2.6. Post Subarachnoid Hemorrhage Vasospasm Treatment

In about 20–30% of patients with subarachnoid hemorrhage the clinical course is complicated by delayed cerebral ischemia (DCI) [73,74]. The underlying mechanisms for DCI are multifactorial, including inflammatory responses, impaired cerebral autoregulation, cortical spreading depolarization and other mechanisms. Diagnosis for triggering treatment of patients at risk for developing DCI is based on multimodal neuromonitoring and vascular neuroimaging modalities [73,74]. In addition to best medical treatment, such as prophylactic administration of a calcium channel blocker (e.g., nimodipine) and optimization of brain perfusion, oxygenation and metabolic demand by hypertensive drugs, endovascular therapy can be attempted in selected patients with symptomatic vasospasm. EVT can be done either by intra-arterial injection of a vasodilating agent or by PTA. Although very high success rates are reported, serious complications, including thrombo-embolism, dissection and vessel rupture, are known to occur [75].

Kamran and Byrne (2015) analyzed FD CT perfusion data sets from 26 patients with aneurysmal subarachnoid hemorrhage, comparing the PBV values with MRI perfusion data sets in the same patients and reference Positron Emission Tomography PET CBV values in literature [18]. They stated that C-arm CT PBV imaging is feasible in patients with DCI after aneurysmal subarachnoid hemorrhage. It appeared that the FD CT PBV values, compared with reported reference CBV values for PET, were relatively high for the white matter and relatively low for the cortical grey matter. They found that FD CT PBV values were a composite perfusion parameter and had preferential (≈60%) blood flow weighting.

A small prospective study of a cohort of six patients confirmed the applicability of FD CT PBV measurement in patients with post subarachnoid hemorrhage vasospasm treated with intra-arterial Verapamil injections [76]. They demonstrated significant increases of blood volumes in treated brain areas post intra-arterial treatment, signifying favorable treatment effect.

**Figure 9:**
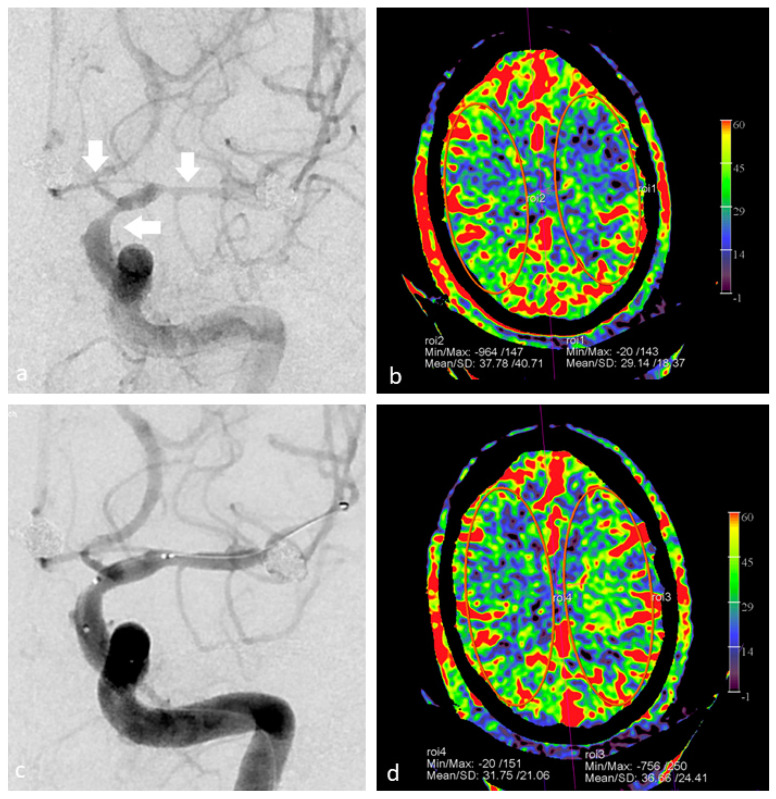
FD CT PBV imaging in a 44-year-old patient after subarachnoid hemorrhage with symptomatic vasospasm. (**a**) DSA in AP view demonstrates severe vasospasm at the level of the left distal internal carotid artery, proximal anterior and middle cerebral arteries (white arrows). (**b**) FD CT PBV mapping with PBV measurements before EVT, displaying lower PBV values at the left hemisphere, compared to the right hemisphere. (**c**) DSA image post dilatation shows favorable angiographic response to PTA. (**d**) Post PTA FD CT PBV measurements show improvement of the PBV value at the left hemisphere compared to the right hemisphere.

Thus, real-time FD CT perfusion imaging in the angiography suite can be of great value, not only for documenting vasospasm and perfusion abnormalities, but also as a diagnostic tool for assessment of intra-arterial treatment response. Besides semiquantitative measurement of angiographic parameters, such as vessel diameters, amount of angiographic staining of the affected brain areas and measurement of color-coded 2D parenchymal perfusion parameters, e.g., contrast bolus arrival time, TTP and MTT, it is possible to compare regional PBV values before, during and after intra-arterial treatment. In this way, proper treatment endpoints of endovascular vasospasm therapy can be established, reducing the risk of procedural complications. In Figure 9, the applicability of FD CT PBV imaging in a patient with post-subarachnoid hemorrhage vasospasm is illustrated.

Concluding, FD CT PBV imaging can be used in diagnosis and treatment evaluation during intra-arterial treatment of post-subarachnoid hemorrhage patients at risk for developing DCI.

### 2.7. Other Potential Indications: Brain Death Determination

FD CT PBV imaging could have a potentially valuable role to document the cessation of blood flow to the brain in brain-dead patients. Evidence-based guidelines exist for determination of brain death in patients with severe neurologic injury [77]. Confirmation of apnea is the mainstay of brain death determination. However, in many situations, e.g., impossibility of adequate clinical evaluation, hypothermia, toxic drug levels, or inconclusive apnea test, additional confirmatory tests are required [77,78]. One of the potential additional tests is determination of absent CBF. This can be done by conventional or MD CT angiography. FD CT perfusion imaging can be performed in the angiography suite without the need for arterial puncture. The cessation of CBF can be determined by demonstrating the absence of contrast filling in cerebral vessels in presence of normal contrast staining of vessels supplied by external carotid arteries.

**Figure 10:**
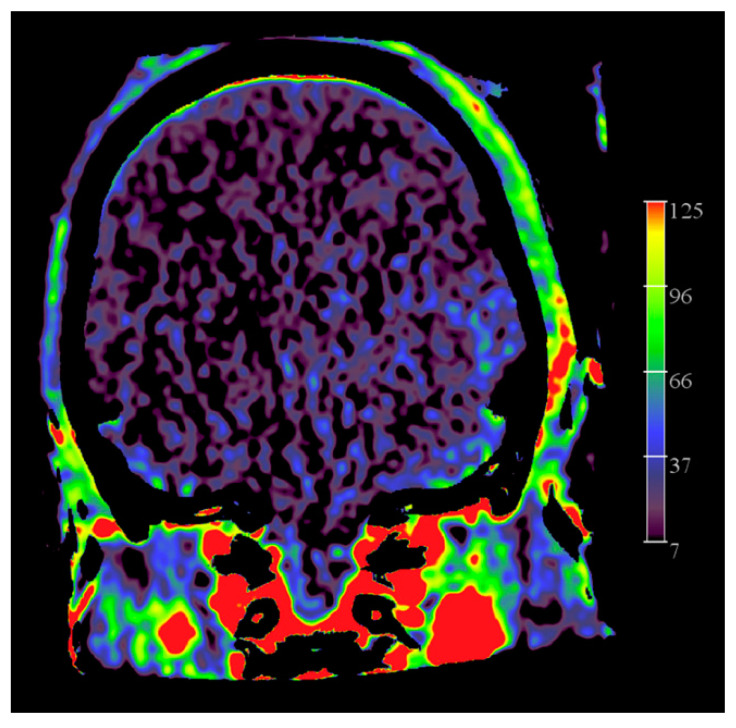
FD CT PBV imaging in a patient with no intracranial flow. Coronal PBV MIP reformation demonstrates filling of the ECA vascular territories, including middle meningeal artery, but no filling of pial arteries.

Figure 10 represents FD CT PBV mapping of a brain-dead patient.

In summary, FD CT PBV imaging can be a potential tool for documentation of cessation of blood flow to the brain in brain-dead patients. However, literature is lacking about the use of this technique in this indication.

## 3. Conclusions

During the past decade, FD CT imaging has enabled real-time imaging of the brain parenchyma and intracranial vessels. Today, the same technique allows us to perform FD CT perfusion imaging in the angiography suite, thereby providing an immediate insight in the brain perfusion of neurovascular patients before, during or after an interventional procedure. Steady-state FD CT PBV imaging is performed by scanning the brain when the intracranial vessels are filled with contrast material. This provides CBV-like PBV mapping images, though the information is different from the parametric maps which are obtained from dynamic perfusion imaging. FD CT perfusion imaging is useful during both diagnostic and interventional neuroradiological procedures. The different methods of contrast injection (intravenous, intra-aortic and selective intra-arterial), as well as the availability of mask and fill runs for evaluation of brain parenchyma and brain vessels on top of semi-quantitative and qualitative analysis of PBV maps, enlarges the armamentarium of the interventionalist in the angiography suite.

The limited number of publications about FD CT PBV mapping, mostly with small groups of patients, emphasizes that the technique is not yet widely accepted or validated. This holds especially true for FD CT PBV imaging of patients with abnormal local or general hemodynamics, which comprise a large proportion of treated patients. Therefore, the exact role of this technique in clinical neuro-interventional practice is yet to be determined, requiring further research. Moreover, the spatial resolution of PBV maps and, in particular the mask run maps, is limited and needs improvement.

A potentially promising perspective is the combination of 3D FD CT PBV imaging with a 2D DSA perfusion imaging. We are confident that future technical improvements, for instance different scan timings and/or contrast injection protocols, improvement of image quality, or implementation of multiphasic scan runs, can make the technique more useful and more accessible for common interventional neuroradiological practice.

The images used in the manuscript were prepared from source images acquired by an Artis Zee with Pure^®^ biplane system (Siemens Healthcare GmbH, Forchheim, Germany) at the angiography unit of the Antwerp University Hospital Radiology department, Belgium.

## Figures and Tables

**Table 1:** Comparison of FD CT PBV perfusion imaging with MD CT perfusion imaging. Approximative values of different aspects of the two imaging modalities are presented. IV = intravenous, IA = intra-arterial.

	FD CT PBV-Perfusion	MD CT-Perfusion
Time of acquisition	12–15 s	60 s
Mode of contrast injection (amount of contrast medium)	IA selective (4 mL)—IA aortic root (25 mL)—IV (60 mL)	IV (45 mL)
Type of parametric mapping	Static (CBV/PBV)	Static and dynamic (CBV-CBF-MTT)
Patient workflow	One-stop-shop	Need for patient transportation

## Data Availability

Not applicable.
